# Homage to James Feeney, a pioneer of biological NMR – Part II

**DOI:** 10.3389/fmolb.2025.1748175

**Published:** 2026-03-05

**Authors:** Gordon C. Roberts

**Affiliations:** University of Leicester, Leicester, United Kingdom

**Keywords:** DHFR, enzymes, large-scale facilities, NMR, structural biology

I was associated with Jim first at Cambridge (1969-72) and then in London (1972–1986). Jim was a pioneer in the application of liquid-state Nuclear Magnetic Resonance (NMR) to chemistry, successively at Liverpool University and Varian Associates (Figure 1), and then, notably, to biomolecules and particularly protein-ligand interactions, where he was a world-leading figure. In 1969 he joined the Medical Research Council (MRC) Molecular Pharmacology Unit in Cambridge, as part of which in 1972 he moved to the MRC National Institute for Medical Research (NIMR) at Mill Hill in London where he worked until retirement, rising to become Head of the Molecular Structure Division and Controller of the Biomedical NMR Centre.

The pioneering and meticulous work of Jim and his team over many years on the binding of ligands to the enzyme dihydrofolate reductase (DHFR), an important drug target, showed the power of solution NMR at its best, probing many intricate details of structure and dynamics in extraordinary depth. His use of ^2^H, ^13^C and ^19^F labelling of both the ligand and the protein demonstrated the ability of NMR to elucidate individual interactions and conformational changes in this system, including the existence of multiple conformations of some enzyme-ligand complexes. With his determination of the solution structures of DHFR-ligand complexes, Jim’s profound understanding not only of NMR but of proteins led to a detailed analysis of the origins of cooperativity in ligand binding to the enzyme. He also employed NMR experiments to obtain detailed information on the dynamics of the complexes, including the making and breaking of individual interactions and correlated rotations of bonds in the ligand and the protein.

**FIGURE 1:**
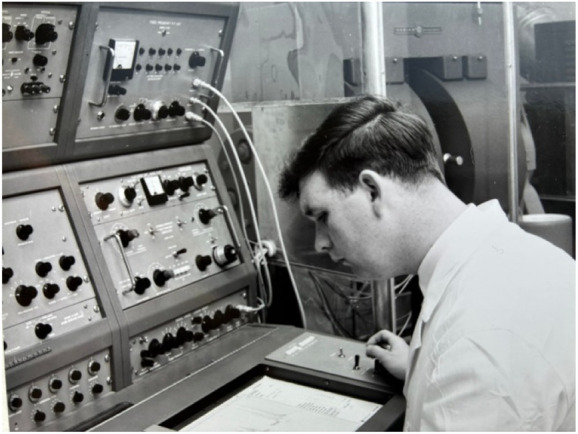
Photo of Jim at an NMR instrument in his early career.

In addition to the work on DHFR for which he is perhaps best known, Jim used NMR to study the structures of oligosaccharides and of the malaria surface protein MSP1 (as part of a project to develop a malaria vaccine) and also the mechanisms of cooperativity in calmodulin and in cyclic AMP receptor protein.

Jim was a genial and friendly man who was always good company. He was notably willing to share not only his deep knowledge of NMR but his enthusiasm for the science, giving support and advice to colleagues over many years. His group members will remember his leading of community singing at Christmas time and, along with their families, will also remember the gatherings at the ‘Mole Pharm’ picnics in the summer. I remember Jim with great affection and respect; he will be sorely missed. In the next paragraphs are the words of other former Mill Hill colleagues.

## Chris Bauer, NIMR, London UK

I worked at NIMR in 1986–1995. When I started work at the NMR Center of NIMR on my first real job, I was very fortunate in having Jim as my boss. Because of his wisdom and intuitive grasp of NMR Jim had a clear view of what needed doing and how it should be done. He made this happen with a genial manner that made working with or for him a joy. It is no surprise that he collaborated with such a large number of people and that visitors from all over the world were drawn to work with him at NIMR.

The NMR Centre was his idea, and it worked so well because he made sure that every user received the maximum benefit from the facilities. This is because he understood what return the MRC were looking for from their generous funding of staff and equipment. Jim not only did groundbreaking research himself but also took the greatest effort to enable others to do so too.

I still remember the anecdotes that Jim used to tell. He delivered them with perfect comic timing. He was also the master of one-liners delivered with a dead-pan expression that just made them so much funnier. That being said, I think I could always detect a subtle change of facial expression when I could see that he was hatching a plan.

I met Jim again at a local tennis club just a few years ago. Although by then he was well into his eighties I could tell from the style of his shots that he must have been a fine player in his time. And of course he was still great company.

## Frederick Benz, University of Louisville, Louisville USA

I worked with Jim in 1970–1972 at the MRC Molecular Pharmacology Unit in Cambridge and then in 1972–1973 at NIMR Mill Hill, London. I was studying for a Ph.D. in Pharmacology at the University of Iowa. My projects primarily involved synthesizing numerous structurally related small molecules and evaluating their biological activity in animals to hopefully but blindly attempt to find the “optimum fit.” At the same time, Dr. Oleg Jardezky at Merck was publishing work on the use of NMR to study protein structure, specifically ribonuclease and its interaction with nucleotides. From reading his papers it was obvious that NMR could provide the “eyes” to convert a blind search for fit into a more informed one. Nearing graduation, I applied to Dr Jardetzy for a post-doc, but he was transitioning his lab to Stanford, had no NMR instruments and suggested I apply to his former post-doc Gordon Roberts at the MRC Molecular Pharmacology Unit in Cambridge, where I met Jim. Having no formal NMR training and looking at the contents of the Emsley, Feeney, and Sutcliffe series, I immediately feared I was in over my head, but Jim and Gordon were very patient tutors. However, without mercy, I badgered Jim with NMR questions, day after day, week after week until exasperated he finally asked me something I have never forgotten; “When are you going to put something into the system.”

Browsing a bookstore, I stumbled across a copy of the newly released, “Pulse and Fourier Transform NMR” by Farrar and Becker. A timely find, as the new 100 MHz Varian XL-100–15 FT NMR instrument had recently arrived. At that time, recording proton NMR spectra in dilute aqueous solution was difficult as the intensity of the residual HDO resonance was 2-3 orders of magnitude greater than the protein or small molecule resonances. Playing around with our new toy with what I had learned from the new FT book, it was possible to minimize or eliminate the residual HDO resonance with appropriate pulse sequences. Alone, I would not have been able to make much of this, but Jim fleshed out the theory and math behind the findings and together with Gordon we published my first NMR paper in J. Mag. Res. in 1972. He generously guided me how to “put something into the system.” His breadth of knowledge, unbounded patience, and unique smirk, which accompanied his wry sense of humor will never be forgotten, nor will the night he got me “tipsy” on scotch one evening at his home.

## Marius Clore FRS, National institutes of health, Bethesda USA

I was in the Division of Molecular Pharmacology NIMR (Mill Hill) from 1980 to 1984. I first met Jim Feeney in December 1978. I was in my final year at UCL Medical School and had written to Sir Arnold Burgen, the then director of the MRC National Institute for Medical Research, a couple of weeks prior with a research proposal to enable me to continue the work I had been doing on low temperature kinetics of cytochrome oxidase that had resulted in several publications. Arnold invited me to come up to Mill Hill by return of mail to discuss. After about 15 min talking, Arnold offered me what was effectively a tenure track position that I would take up in August 1980 once I had completed my house jobs. But Arnold told me that he did not have the funds for the equipment I would need. He suggested I do NMR instead in the Division of Molecular Pharmacology as he figured NMR would be right up my alley. He then handed me over to Jim who first took me over to HR to complete and sign a contract on the dotted line (something that would be unthinkable these days) and then proceeded to show me around, including, of course, the NMR facility which at the time consisted of a Bruker 270 MHz spectrometer. Eighteen months later, I showed up at Mill Hill and Jim again showed me around and my lab and tiny office (probably about 6 square feet) that I was to share with Angela Gronenborn. The rest, so they say, is history. Jim was a great colleague. I would run ideas off him and shoot the breeze almost every day, in addition to our interactions at lunchtime and, in characteristic English tradition, twice a day at tea time. Jim was an excellent person to bounce ideas off, as he would always think critically about a problem, and most importantly a genuinely nice and kind person. When I left in 1984 to head the Biological NMR group at the Max Planck Institute for Biochemistry in Martinsried Germany, I kept in frequent touch with Jim. After I moved to the NIH in 1988, we did not communicate as much but remained in touch. The last time I saw Jim in person was in 2010 at a meeting in London at Imperial College to which he came especially to hear me give the RSC Centenary Prize lecture, and the last time I communicated with Jim by email was in 2021. He was still very sharp and interested in what I was doing. Jim’s passing is a great loss and truly marks the end of an era.

## Mark Forster, NIMR, London UK

The National Institute for Medical Research (NIMR) at Mill Hill was a hotbed of biological research for many decades. The MRC biomedical research centre was led by Jim Feeney, when I started working there in 1984, Jim was always welcoming, supportive and encouraging. His warm personality and knowledge, making him the best type of manager one could have. My knowledge of macromolecular NMR structure and dynamics grew greatly in this fantastic environment.

I recall Jim as an excellent singer, for example, at the NMR conferences that we attended. He also created and performed many witty tunes for our NIMR Christmas shows. I have always remembered this episode when we were visited by the salesman from the mighty Silicon Graphics corporation. During the sales pitch the skilled and slick salesman came up with the phrase - “With this workstation, the only limit is your imagination”. Keeping a straight face Jim looked at the salesman and replied “Oh dear, my imagination is very limited”:-) The slick sales pitch sputtered and stalled under some very dry humour. I smirked, it was classic Dr Feeney. In the end we did purchase the workstation - a Silicon Graphics 3,020 as I recall. For me it was the beginning of an epic journey. Learning the Unix command line was life enhancing in several ways, because of the doors it opened for me. Those are skills which remain valuable to this day, thanks to Jim.

## Gill Ostler, NIMR, London UK

It was with great sadness to be told by Jim’s daughter Cathy, of the death of her Father. I was working at the Institute at Mill Hill long before Jim came from Cambridge in 1972 with Sir Arnold Burgen’s group. During the 23 years that I spent working in Jim’s group at the Institute in Mill Hill, I found him to be an amazing boss, a Father figure to us all, always approachable, very supportive even when things were not going as planned and looking on the positive side when I took my problems to him. Beside all this, he had a wonderful sense of humour, not only seen everyday in the work place but it came into its own in the sketches and songs that he wrote for our Christmas Shows. It has been a great privilege and pleasure to have known Jim and his lovely family and he leaves behind so many lasting, wonderful memories of someone who will be sorely missed by all who were fortunate to know him.

